# A Multicenter Randomized Controlled Trial of Measurement-Based Care for Major Depressive Disorder in Pakistan

**DOI:** 10.1192/bjo.2024.123

**Published:** 2024-08-01

**Authors:** Zahra Nigah, Nasim Chaudhry, Nusrat Husain, Imran Chaudhry, Ishrat Husain

**Affiliations:** 1 Pakistan Institute of Living & Learning, Multan, Pakistan; 2 Pakistan Institute of Living & Learning, Karachi, Pakistan; 3 Dow University of Health Science, Karachi, Pakistan; 4 University of Manchester, Manchester, United Kingdom; 5 Centre for Addiction and Mental Health, Toronto, Canada; 6 Department of Psychiatry, University of Toronto, Toronto, Canada

## Abstract

**Aims:**

A worldwide public health concern is major depressive disorder (MDD) with limited availability and access to evidence-based treatment in low- and middle-income countries (LMICs) such as Pakistan. Measurement-based care (MBC) is a low-cost strategy to improve clinical outcomes for people with MDD that involves the systematic administration of validated outcome measures to inform treatment decisions. However, research on MBC's effectiveness in LMICs is scarce. This paper aims to evaluate the feasibility and clinical effectiveness of MBC against standard care for patients with moderate to severe MDD in Pakistan.

**Methods:**

This is a multicenter randomized control trial. Participants (n = 154) of 18 to 65 years of age recruited from psychiatric units of teaching and non-teaching hospitals and primary care settings such as General Physician (GP) clinics and Basic Health Units (BHUs) from 6 cities were randomised to receive MBC (guided by a schedule), or standard treatment (guided by clinicians’ judgement). Patients were prescribed by treating clinicians either with mirtazapine (7.5–45 mg/day) or paroxetine (10–60 mg/day) for a period of 12 weeks. All participants, regardless of their treatment arm, were followed-up till 24 weeks post-randomization and assessed for severity of depression. Side effects were regularly monitored using standard checklist. Outcome assessors were blind to treatment allocation.

**Results:**

The Pakistani National Bioethics Committee (NBC) has granted complete ethical approval. A total of 15 psychiatrists and 4 General Practitioners (GPs) were approached and invited to participate in the study and consent was given by 9 psychiatrists and 2 GPs. A total of 351 patients were screened against eligibility criteria and 177 were eligible to participate. A total of 154 eligible participants consented (87%) to participate and were recruited and randomized into the trial. A total of 131 randomized participants (85%) completed 24-month follow-up. Only two adverse events were reported during the trial period. Recruitment, retention and safety analysis indicates feasibility of the trial in Pakistani healthcare context. The data are being analyzed for effectiveness outcomes.

**Conclusion:**

It is essential to investigate the viability, usefulness, and efficacy of MBC for MDD in low-resource settings due to mounting data from high-income settings confirming its effectiveness. The planned trial's outcomes may help build a scalable, low-cost method for effectively improving outcomes for MDD patients in Pakistan.

